# La neurobrucellose: une cause curable de surdité neurosensorielle à ne pas méconnaitre

**DOI:** 10.11604/pamj.2015.22.122.7671

**Published:** 2015-10-12

**Authors:** Alae Bezzari Malhi, Mohamed Ridal, Siham Bouchal, Mohammed Faouzi Belahsen, Mohamed-Nourdine El Alami

**Affiliations:** 1 Service d'Oto-Rhino-Laryngologie, Centre Hospitalier Universitaire Hassan II, Fès, Maroc; 2 Service de Neurologie, Centre Hospitalier Universitaire Hassan II, Fès, Maroc

**Keywords:** Neurobrucellose, surdité, méningite, sérologie de Wright, neurobrucellosis, hearing loss, meningitis, Wright serology

## Abstract

La brucellose est une zoonose ubiquitaire touchant en particulier les pays méditerranéens et le Moyen-Orient. Les manifestations neurologiques sont assez diverses. Nous rapportons l'observation d'un patient âgé de 45 ans, agriculteur, ayant consulté pour une surdité profonde bilatérale depuis 2 mois associée à des céphalées, des épisodes d'hémiparésie à bascule, de trouble de langage, spontanément résolutifs en quelques minutes et des sensations vertigineuses intermittentes depuis 09 mois. Les résultats de la ponction lombaire, de l'imagerie par résonnance magnétique et surtout la sérologie ont permis de conclure à une neurobrucellose. L’évolution sous bi-antibiothérapie a été favorable, avec régression des signes neurologiques, normalisation du LCR et amélioration de la surdité. La neurobrucellose est une affection grave dont le pronostic dépend de la précocité du diagnostic et du traitement. Nous pensons qu'un tableau clinique associant une surdité neurosensorielle et une symptomatologie neurologique progressive doit évoquer en premier une neurobrucellose, d'autant plus que le patient est à risque et dans les pays où cette maladie est endémique.

## Introduction

La brucellose est une zoonose ubiquitaire touchant en particulier les pays méditerranéens et le Moyen-Orient. Son incidence est en nette régression dans les pays développés alors qu'elle pose toujours un problème de santé publique dans les pays en voie de développement. Les manifestations neurologiques de la brucellose sont polymorphes et souvent plurifocales chez un même patient. L'atteinte de la VIIIe paire crânienne est fréquente, pouvant être la manifestation clinique majeure de la maladie. Nous présentons un cas de surdité bilatérale profonde neurosensorielle associée à des céphalées et des anomalies du signal de la substance blanche à l'IRMayant conduit au diagnostic de neurobrucellose.

## Patient et observation

Un homme âgé de 45 ans, agriculteur, a consulté pour une surdité profonde bilatérale depuis 2 mois associée à des céphalées modérées temporo-frontales. Le patient présente dans ses antécédents une otospongiose opérée en 1990, avec des aphtes buccaux à répétition. L'interrogatoire trouve des épisodes d'hémiparésie à bascule, de trouble de langage, spontanément résolutifs en quelques minutes avec des sensations vertigineuses intermittentes depuis 09 mois.

**Figure 1:**
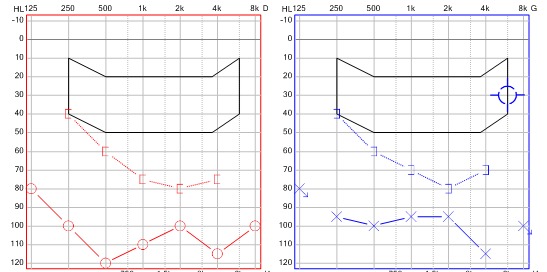
Audiométrie initiale confirmant la présence d'une surdité bilatérale ainsi que sa nature perceptionnelle

L'examen clinique trouvait un patient conscient, bien orienté, apyrétique, sans syndrome méningé et avec un examen neurologique sans particularités. L'audiométrie avait objectivé une surdité mixte à prédominance perceptionnelle (Figure 1). Il n'existait, en revanche, pas de signes d'atteinte vestibulaire, avec une vidéonystagmographie sans signes d'atteinte centrale.

**Figure 2:**
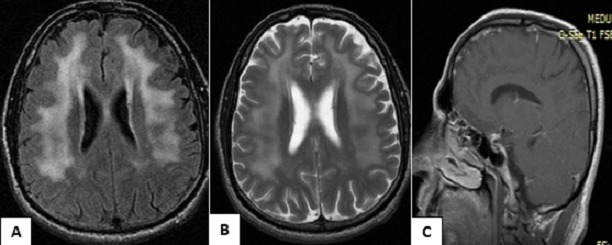
IRM cérébrale coupes axiales séquence Flair (a) et T2 (b) montrant un hypersignal péri-ventriculaire (leuco-encéphalopathie). La séquence T1 injectée en coupe sagittale (c) montrant une prise de contraste méningée nodulaire

L'IRM cérébrale avait objectivé des lésions en plage de la substance blanche bilatérales autour des ventricules latéraux, hyperintenses en séquences pondérés T2 et Flair, avec une prise de contraste leptoméningée, sans image anormale dans la fosse cérébrale postérieure et les nerfs auditifs paraissaient normaux (Figure 2).

Les examens biologiques ont révélé un syndrome inflammatoire et l'ionogramme était sans anomalie notable. La ponction lombaire montrait une méningite à 400 éléments blancs/mm^3^ à prédominance lymphocytaire, une protéinorrachie élevée à 1,9g/l, avec une culture négative. Les sérologies du VIH, de la syphilis, des hépatites B et C, de la maladie deLyme ainsi que la sérologie de Wright (brucellose) étaient négatives. Un bilan cardiovasculaire et immunologique s'est révélé également sans anomalie notable. Devant la symptomatologie du patient, sa profession et les résultats de la ponction lombaire, l'ensemble faisant suspecter une neurobrucellose, une autre technique de sérodiagnostic a été réalisée: la réaction de fixation du complément, qui s'est révélée positive dans le sang et le liquide céphalo-rachidien, avec des titres de 1/64 et 1/128 respectivement.

**Figure 3:**
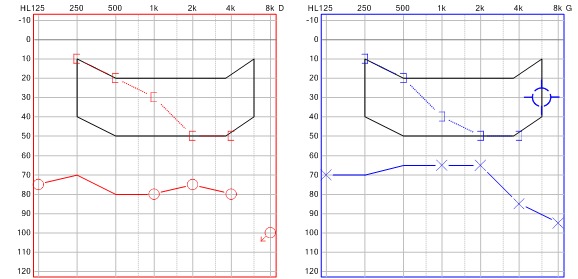
Audiométrie de contrôle à 6 mois montrant l'amélioration de la surdité (noter l'amélioration des courbes osseuses)

Le diagnostic de la neurobrucellose, type méningo-vascularite,fut retenu et une bithérapie anti-brucellienne associant Doxycycline (200 mg/j) et Ciprofloxacine (500 mg/j) fut initiée et poursuivie pendant 6 mois. Un traitement adjuvent par corticothérapie a été associé durant les 6 premières semaines. L’évolution a été favorable avec régression complète des signes neurologiques, des céphalées, normalisation du LCR et amélioration satisfaisante de sa surdité objectivée sur une audiométrie de contrôle à 6 mois (Figure 3).

## Discussion

La brucellose est une zoonose majeure fréquente dans le monde, touchant plus de 500 000 cas par an. Le germe qui en est responsable est un Gram négatif appartenant au genre *Brucella*. Plusieurs espèces sont décrites dont les plus fréquentes sont *B. abortus* et *B. melitensis*. La prévalence de la maladie peut atteindre dans les pays méditerranéens 10/100 000 habitants [1]. C'est une maladie à déclaration obligatoire reconnue comme maladie professionnelle pour les individus au contact de ruminants infectés ainsi que pour le personnel de laboratoire [2]. La transmission à l'homme se fait principalement par contact direct avec le bétail, en général par voie cutanéomuqueuse ou indirectement par voie digestive; la contamination est alors liée aux habitudes alimentaires (lait cru, fromage frais, crème non pasteurisée). La contamination interhumaine est exceptionnelle [3].

Les manifestations cliniques de la brucellose sont peu spécifiques. Elles peuvent survenir sur un mode aigu, ou être plus insidieuses. Elle se manifeste le plus souvent sous forme d'un tableau pseudogrippal: fièvre, sueurs, anorexie, asthénie, céphalées, myalgies, arthralgies. Sa forme classique « sudoro-algique » est rarement observée actuellement. L’évolution spontanée de la brucellose se caractérise surtout par la possibilité de survenue de localisations secondaires qui font la gravité de la maladie. Celles-ci peuvent être notamment neuroméningées, cardiaques, ostéoarticulaires, hépatospléniques, ou génitales [3]. Les formes chroniques se définissent par une évolution prolongée au-delà d'un an, avec ou sans découverte d'un foyer infectieux focalisé [4].

L'atteinte du système nerveux est rare dans la brucellose avec une grande diversité des modes de présentation clinique[5]. La fréquence des manifestations neurologiques de la brucellose (neurobrucellose) ne dépasse pas 10% des cas [3, 5]. Ilpeut s'agir de méningite, de méningo-encéphalite aiguë ou chronique, d'hypertension intracrânienne, de méningomyélite, de compression médullaire par spondylodiscite, de syndrome cérébro-vasculaire,d'atteinte de nerfs crâniens ou de polyradiculonévrite.

Les critères nécessaires pour retenir l'atteinte neurologique au cours d'une brucellose sont: la présence de signes cliniques neurologiques, des anomalies du LCR avec prédominance lymphocytaire et hyperprotéinorrachie, la mise en évidence du germe au niveau du LCR après culture ou sérologie brucellienne positive, et enfin une réponse favorable après antibiothérapie avec baisse du taux de lymphocytes et des protéines au niveau du LCR [6]. Dans notre cas, trois des quatre critères sus-cités ont été présents. L'hypoacousie neurosensorielle semble être un signe fréquent de la méningoencéphalite brucellienne chronique (19 cas d'hypoacousie dans la série de 23 cas d'Al-Sous et al) [7]. Le tropisme particulier de cette maladie pour les voies auditives est confirmé par plusieurs publications [5, 6, 8, 9]. Cela dominait le tableau clinique chez notre patient. Yacub et al. avaient même trouvé des anomalies des potentiels évoqués auditifs chez leurs huit patients atteints de méningite brucellienne [10].

Les anomalies en imagerie par résonnance magnétique (IRM) du système nerveux central le plus souvent observées dans la neurobrucellose sont de trois types: lésions inflammatoires, anomalies de signal de la substance blanche et lésions vasculaires ischémiques [5]. L'IRM de notre patiente avait montré un hypersignal de la substance blanche avec prise de contraste méningée traduisant son inflammation.

L'isolement des Brucella en culture est la technique de référence pour établir un diagnostic certain de brucellose [3]. Cet isolement se fait habituellement à partir du sang, par hémoculture, ou plus rarement à partir d'autres prélèvements en fonction du contexte clinique. Les hémocultures sont souvent contributives à la phase aigüe de la maladie, moins à la phase subaiguë ou chronique. La PCR est possible mais reste réservée à certains laboratoires, elle est particulièrement utile en cas d'antibiothérapie ayant précédé les analyses.

Les méthodes sérologiques sont nombreuses. La technique de séroagglutination de Wright est la première technique sérologique décrite, et demeure la référence préconisée par l'Organisation Mondiale de la Santé du fait de sa standardisation. Les autres techniques sérologiques développées incluent notamment la technique d'agglutination sur lame ou épreuve de l'antigène tamponné, la réaction de fixation du complément, la technique d'immunofluorescence indirecte, et les tests Elisa. Quelque soit le test sérologique, des réactions croisées sont observées avec des infections par Yersinia enterolitica O9, Vibriocholerae et Francisellatularensis.

Le traitement de la neurobrucellose doit être précoce, basé sur des antibiotiques ayant une bonne diffusion à travers la barrière hématoencéphalique et en intracellulaire. Les antibiotiques les plus actifs sont les aminosides (streptomycine et gentamicine), les tétracyclines, la rifampicine, et les fluoroquinolones [3]. L'association d'au moins deux antibiotiques est la règle pendant une durée minimum de trois mois [1]. Les deux associations les plus utilisées sont: Rifampicine/Triméthoprime'Sulfaméthoxazole ou Rifampicine/Doxycycline [5]. Pour notre cas, on n'a pas pu utiliser la rifampicine étant donné que cette molécule est réservée au traitement de la tuberculose dans notre pays. Le meilleur traitement de la brucellose est préventif par le contrôle et l’élimination de l′infection chez les animaux (surveillance sérologique, abattage des animaux infectés et vaccination des jeunes animaux) ainsi que la pasteurisation du lait. Il n'existe pas de vaccin à usage humain.

## Conclusion

La diversité clinique et les complications engendrées par la brucellose rendent son diagnostic clinique difficile. Nous pensons qu'un tableau clinique associant une surdité neurosensorielle et une symptomatologie neurologique progressive doit évoquer en premier uneneurobrucellose, d'autant plus que le patient est à risque et dans les pays où cette maladie est endémique.

## References

[1] Pappas G, Papadimitriou P, Akritidis N, Christou L, Tsianos EV (2006). The new global map of human brucellosis. Lancet Infect Dis..

[2] Janbon F (2000). Brucellose. EMC - Maladies Infectieuses.

[3] Maurin M (2005). La Brucellose à l'aube du 21e siècle. Med Mal Infect..

[4] Young EJ (1995). An overview of human brucellosis. Clin Infect Dis..

[5] Awada A, Korri H, Issa Z, Ali Y, Beaini M (2011). Paraparésie et surdité progressives avec leuco-encéphalopathie révélant une neurobrucellose chronique. Revue neurologique..

[6] Showkat HI, Sarmast AH, Lone L, Hussain I, Kotwal S (2012). Neurobrucellosis with bilateral sensorineural hearing loss and ataxia a case report. Schweizer archive fürneurologie und psychiatrie.

[7] Al-Sous MW, Bohlega S, Al-Kawi MZ, Alwatban J, McLean DR (2004). Neurobrucellosis: clinical and neuroimaging correlation. AJNR Am J Neuroradiol..

[8] Aysha A, Alshareef MD (2009). Case Report of Polyradiclopathy, Hearing Loss, and Ataxia as Presentation of Neurobrucellosis; JKAU. Med Sci..

[9] Valenza G, Kallmann B, Berend A, Mlynski R, Nöckler K, Kurzai O, Frosch M, Abele-Horn M (2006). Isolation of Brucellamelitensis from a patient with hearing loss. Eur J Clin Microbiol Infect Dis..

[10] Yacub B, Kabiraj MM, Shamena A, Al-Bunyan M, Daif AK, Tahan A (1992). Diagnostic role of brain-stem evoked potentials in neurobrucellosis. Electroencephalogr Clin Neurophysiol..

